# Reduced migration of esophageal fully covered self-expandable metal stents affixed with clips with anchor prongs

**DOI:** 10.1016/j.igie.2025.01.005

**Published:** 2025-01-17

**Authors:** Jason DuBroff, Daniel Holten, Gregory Toy, David Jonason, Daryl Ramai, John Morris, John Fang, Christopher Ko

**Affiliations:** Division of Gastroenterology, Hepatology and Nutrition, University of Utah Health, Salt Lake City, Utah, USA

## Abstract

**Background and Aims:**

Fully covered self-expandable metal stents (FCSEMSs) are a common therapeutic treatment for benign and malignant esophageal disorders. Stent migration is a common adverse event of FSCEMSs. We describe clips with anchor prongs (CAPs) as a novel technique to reduce FCSEMS migration.

**Methods:**

We retrospectively analyzed 27 patients with FCSEMSs affixed with CAPs and 28 patients without FCSEMS affixation for stent migration as the primary endpoint and used previously published data to determine the relative risk reduction (RRR).

**Results:**

Migration was observed in 14.8% of cases (RRR, 43%). CAP affixation was associated with a decreased odds of migration (odds ratio, .19; *P* = .02). The only adverse event reported in those with (n = 4) or without (n = 14) CAP fixation was postprocedural pain.

**Conclusions:**

CAPs may reduce stent migration, providing a novel option for the endoscopist seeking a means of securely attaching esophageal FCSEMSs.

Fully covered self-expandable metal stents (FCSEMSs) are a common therapeutic intervention for benign and malignant esophageal disorders such as refractory strictures, perforations, fistulas, foregut surgical adverse events. and palliation of esophageal carcinoma.^1^ Migration is a well-known adverse event of FCSEMS placement,^2^ which provides opportunities for novel antimigratory techniques and technologies. Current endoscopic techniques for FCSEMS fixation include endoscopic suturing, over-the-scope clip (OTSC) fixation systems, and conventional through-the-scope (TTS) clips, which are all associated with lower migration rates than unaffixed FCSEMSs.^3^ We examined the efficacy of novel TTS clips with anchor prongs (CAPs; MANTIS; Boston Scientific, Marlborough, Mass, USA) for FCSEMS fixation to minimize stent migration.

## Methods

A retrospective chart review was performed for patients who had placement of FCSEMSs, either unaffixed or affixed with CAPs, with migration as a primary endpoint from January 2019 to January 2024. Migration was defined as endoscopic or imaging findings confirming that the stent was out of position as compared with the initial placement and was identified either because of clinical symptoms concerning for stent dysfunction or at the time of follow-up endoscopy.

**Figure 1:**
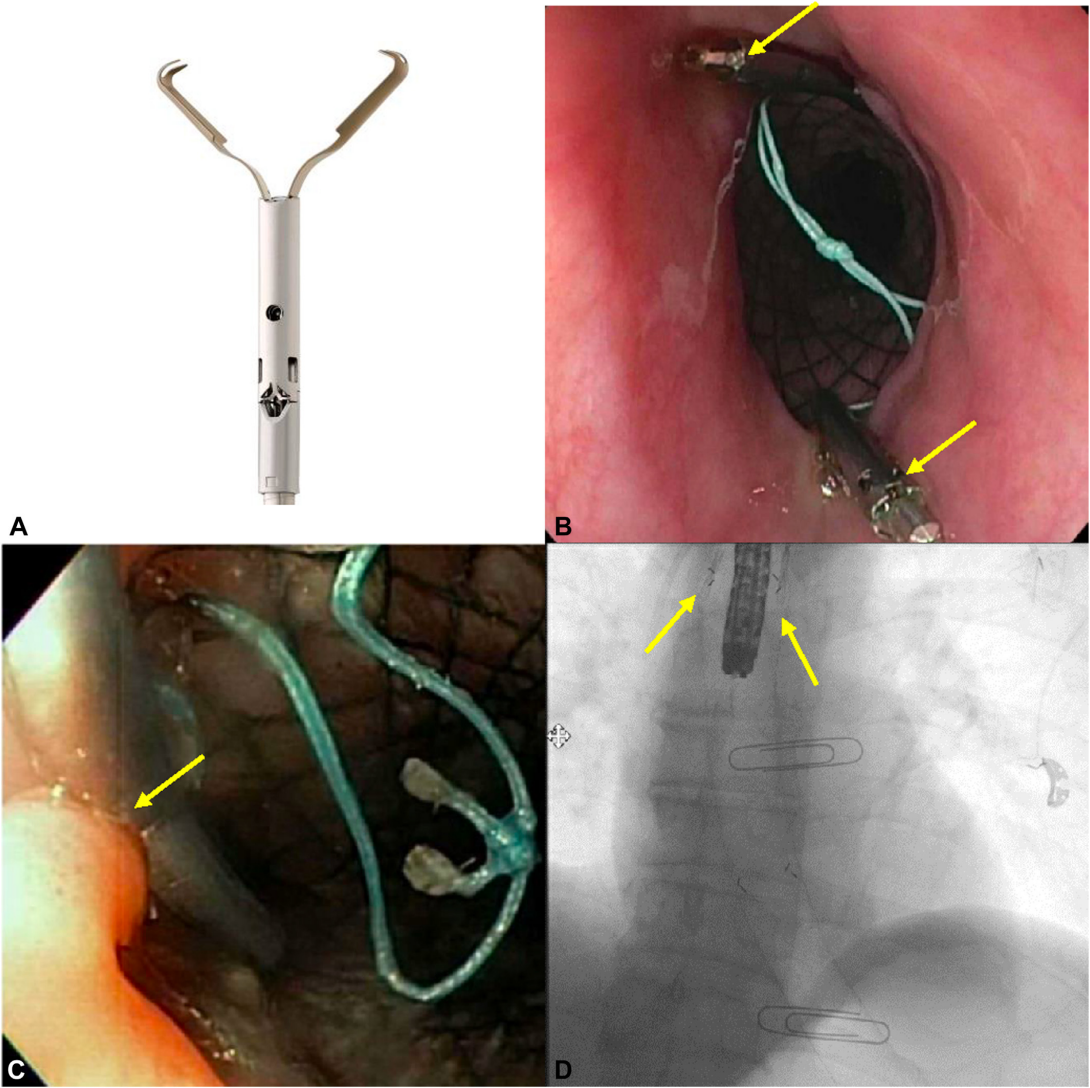
**A,** Clip with anchor prongs (CAPs). **B,** Two CAPs (indicated with arrows) used to secure the esophageal stent. **C,** Mucosal crease (indicated with arrow) after successful CAP fixation. **D,** Fluoroscopy of esophageal stent with 2 CAPs (indicated with arrows).

Twenty-seven patients were identified as having undergone esophageal FCSEMS placement affixed with CAPs, whereas 28 control subjects with unaffixed FCSEMS placement were identified over the same study period. The endoscopic technique for CAP stent fixation encompassed using 1 arm of the CAPs to create a crease in the esophageal mucosa and then deploying after the second arm made purchase with the proximal end of the FCSEMS (Fig. 1).

The primary outcome of interest was any stent migration before interval endoscopy follow-up or, if long-term stent placement was planned, within 3 months after the initial placement, whichever resulted in a shorter interval. Additional covariates were age, sex, date of stent placement, indication, presence of any stricture, presence of esophageal malignancy, number of follow-up procedures over 1 year, use of a TTS stent, location of stent placement, number of CAPs used, and stent longevity (Table 1). Longevity was defined as the number of days until planned or unplanned stent removal. Patients were also assessed for postprocedural adverse events such as postprocedural pain, bleeding, and perforation.

**Table 1:** Characteristics of esophageal FCSEMSs affixed with CAPs

Characteristics	FCSEMSs with CAP affixation (n = 27)	FCSEMSs without CAP fixation (n = 28)
Mean age, y	60.2	60.5
Female sex	41 (11)	36 (10)
Migration events (over 3 mo)		
Overall∗	14.8 (4)	25 (7)
Through-the-scope stent	7.4 (2)	7.1 (2)
Malignant stricture	3.7 (1)	17.9 (5)
Any stricture (malignant and benign)	7.4 (2)	32 (9)
Mean follow-up esophagogastroduodenoscopy after placement (<12 mo)∗	1.14	2
Through-the-scope stent deployment	59.3 (16)	25 (7)
Stent longevity, days		
Mean	42.3	57.7
Median	43	43
No. of CAPs		
Mean	2.3	0
Median	2	0
Indication∗		
Surgical/anastomotic leak	44.4 (12)	10.7 (3)
Benign stricture	3.7 (1)	21.4 (6)
Esophageal perforation	14.8 (4)	10.7 (3)
Malignant stricture	29.6 (8)	53.6 (15)
Other	11.1 (3)	3.5% (n=1)
Adverse events		
Perforation	0	0
Bleeding	0	0
Postprocedural pain∗	14.8 (4)	50 (14)

Values are % (n) unless otherwise defined.

*FCSEMS*, Fully covered self-expandable metal stent; *CAP*, clip with anchor prong.

∗ Significant finding (*P* < .05).

Relative risk reduction (RRR)∗ was calculated using previously published data on unaffixed FCSEMS migration. Data analysis was performed using the χ^2^ test, logistic regression, and the Student *t* test. Univariate analysis was performed to assess for significant relationships. All data were analyzed using Stata (StataCorp, College Station, Tex, USA). Institutional review board approval was obtained. This research received no specific grant from public or private sectors.

## Results

Mean patient age was 60.6 years, and 38% were women. Technical success was achieved in 100% of cases. Stent migration in patients with CAPs was observed in 4 cases (14.8%) overall (Table 2). To evaluate RRR, a migration rate of 26% was selected from a large (n = 369) multicenter study that, like our study, included both benign and malignant indications, used a similar variety of stents, and also drew from patients at our own institution.^4^ Using this previously published combined migration rate of 26%^4^ revealed an RRR of 43% among FCSEMSs affixed with CAPs in our study. One migration event was believed to be related to an unsuccessful 2-hour attempt at feeding tube placement by the primary team after stent placement. Exclusion of this aberrant case yielded an RRR of 57.3%.

**Table 2:** Migration rates between unaffixed FCSEMSs and FCSEMSs affixed with CAPs and calculated relative risk reduction

	FCSEMS affixed with CAPs	Unaffixed FCSEMSs
Migration rate, %	14.8	26
Relative risk reduction,∗ %	43	

*FCSEMS*, Fully covered self-expandable metal stent; *CAP*, clip with anchor prong.

∗ Relative risk reduction calculated as the difference between unaffixed migration and CAP affixed migration rate divided by unaffixed migration rate.

Migration was not significantly associated with strictures (benign or malignant), use of TTS stent deployment, stricture severity, or stent location. Overall migration among unaffixed control subjects was observed in 25% of patients (n = 7). A significant association was found between migration and indication for stent placement (*P* = .02) as well as use of CAPs (*P* = .02). Univariate logistic regression showed a decreased odds of migration among stents affixed with CAPs (odds ratio, .19; 95% confidence interval, .05-.79; *P* = .02). Adjusting for indication, the presence of strictures (benign and malignant) and use of TTS stents did not affect the significance of CAP fixation on stent migration.

There was no difference in overall stent longevity between the study groups. However, stents affixed with CAPs had, on average, a fewer number of follow-up endoscopies over 1 year (1.13 vs 2 endoscopies, *P* = .01). Our initial primary endpoint of stent migration within 3 months of placement was adequate to ensure all migration events were captured. Accordingly, we found the median stent longevity (ie, 43 days for CAP fixation and 32 days for no fixation) was substantially shorter than 3 months.

Postprocedural pain was the only reported adverse event among those with (n = 4) and without (n = 14) CAP fixation. Stents affixed with CAPs were associated with a decreased odds of postprocedural pain (odds ratio, .17; 95% confidence interval, .05-.58; *P* < .01).

## Discussion

Migration of esophageal FCSEMSs is a known and common adverse event for both benign and malignant esophageal indications. This study shows that FCSEMSs affixed with CAPs have decreased migration rates when compared with unaffixed stents and may be associated with fewer follow-up endoscopies and less postprocedural pain. TTS stents are typically associated with higher migration rates because of a weaker radial force,^5^ but these stents did not affect the significance of CAP fixation on migration in this study.

The significant reduction in postprocedural pain was an unexpected finding. We suspect that CAP fixation may pull the esophageal wall toward the stent rather than the stent pushing against the esophagus, thus lowering the radial force exerted on the esophagus and resulting in less pain. However, additional studies are needed to further describe this association.

CAPs are currently used for large (<3 cm) defect closures typically after endoscopic mucosal resection or endoscopic submucosal dissection because the anchor-pronged design and strong grasping force allow for deep and secure mucosal approximation of tissue edges. These anchor prongs may also provide desirable properties for short-term fixation of FCSEMSs that may result in fewer migrations without restricting stent removal if required.

The efficacy of stent fixation using CAPs as compared with OTSCs warrants future investigation. A 2022 retrospective study of 433 procedures found a lower rate of migration with OTSC stent fixation (up to 35%) when compared with unaffixed FCSEMSs and suturing,^6^ which is higher than the 14.8% observed in this analysis.

Compared with other methods, fixation with CAPs is facile because clips are deployed under direct visualization TTS, allowing reorientation and repositioning. There is no need to remove the endoscope from the patient, use proprietary removal devices, or delay placement because of the setup process as with other methods. Additionally, CAP (unit price, $350) fixation is a cost-effective option given that OTSCs typically cost $1000.^7^ Our analysis indicates CAP fixation may reduce the average number of repeat endoscopies, which further reduces costs.

This study is limited by its small sample size because CAPs only recently received approval from the U.S. Food and Drug Administration in 2022, restricting available cases. Larger prospective studies are needed to investigate the efficacy of CAP fixation in preventing FCSEMS migration with direct comparison with currently used fixation methods. CAPs have already been reported for other uses that require closure and secure attachment.^8^ This study is the first to describe a promising, novel application of CAPs that is readily available for endoscopists wishing to reduce FCSEMS migration.

## Disclosure

The following author disclosed financial relationships: J. Fang: Consultant for Merit, Circa Scientific, and Aspero Medical. All other authors disclosed no financial relationships.

## Footnotes

∗ Relative risk reduction is defined as the difference between the control event rate and experimental event rate divided by the control event rate.
